# Case Report: Rare site for intraoral meningioma

**DOI:** 10.12688/f1000research.21999.2

**Published:** 2020-04-06

**Authors:** Hatem Wael Amer, Layla Hafed, Sally Ibrahim, Shady Shaker

**Affiliations:** 1 Faculty of Dentistry, Cairo University, Cairo, Egypt; 2 Faculty of Dentistry, Ahram Canadian University, Cairo, Egypt; 3 Faculty of Dentistry, El-Fayoum University, Cairo, Egypt

**Keywords:** Intra-oral meningioma, Benign tumor, Ectopic meningioma, Palatal lesion

## Abstract

Extracranial meningioma is very rare with few cases reported, especially in the oral cavity. Its diagnosis is considered a challenge owing to the unusual site of occurrence.  We report, to the best of our knowledge, the first case of extra-cranial meningioma as a primary tumor in the hard palate with no detected intracranial extension. A 59-year-old Egyptian female patient presented with a 22-year history of a large painless swelling at the right side of the hard palate, which could not be seen on radiographs.  An incisional biopsy was taken and, after assessment with a panel of immunohistochemical markers, the lesion was diagnosed as extracranical grade I mengiothelial meningioma. The patient did not show up for surgical excision and follow-up was not performed because of the lose of contact with the patient. Intraoral meningioma is a rare tumor. Immuohistochemical markers are important for confirming this diagnosis.

## Introduction

Meningioma is a benign neoplasm of meningothelial cells ^1^. Meningioma may develop as a direct extension of a primary intra-cranial meningioma or as a true primary extra-cranial meningioma ^2^.

Extra-cranial (ectopic) tumors are mostly seen in the head and neck region with no connection intra-cranially ^3^. The most common extra-cranial site is the orbits. Meningioma arising in the oral cavity is extremely rare ^4^. To the best of our knowledge, 19 cases have currently been reported in the oral cavity ^2, 4– 20^ and we are reporting the first case in the hard palate.

## Case report

**Figure 1.**
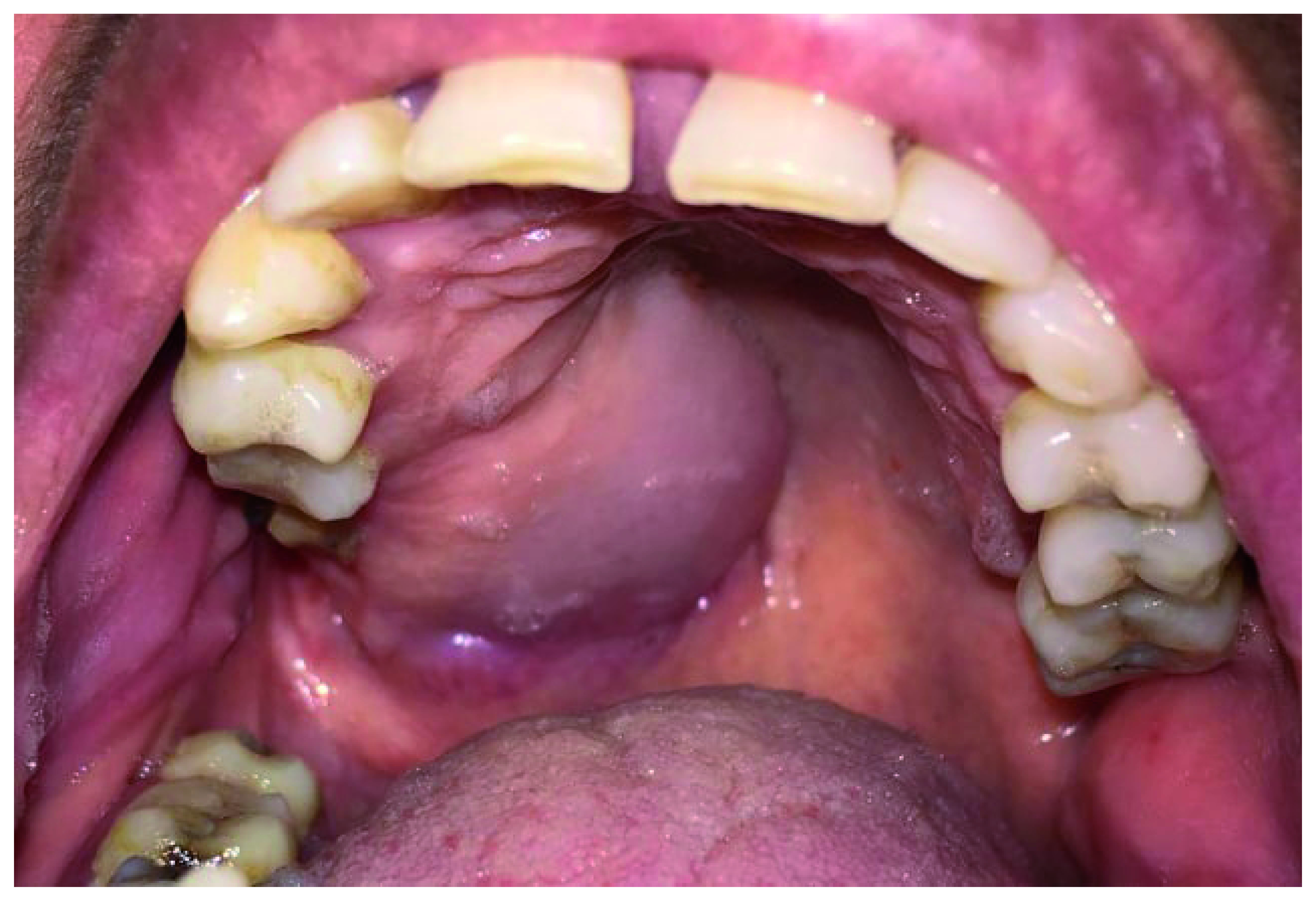
Preoperative clinical picture showing 3 × 3cm swelling in the hard palate.

A 59-year-old female patient presented to the outpatient clinic in the Oral and Maxillofacial Surgery Department, Cairo University in January 2019 complaining of a large painless swelling in the hard palate ( Figure 1). The patient reported that the swelling had been present in her oral cavity for 22 years. The patient’s medical and familial histories were unremarkable. As well as there was not a history of exposure to radiation. Upon clinical examination on the day of admission, a large hard palatal swelling (3 cm × 3 cm) was evident on the right side of the hard palate. The swelling was covered by normal mucosa and showed a slight bluish tinge. A provisional diagnosis of a benign peripheral nerve neoplasm and a minor salivary gland benign neoplasm were made. CT scan was performed with no evidence of bone involvement. 

**Figure 2.**
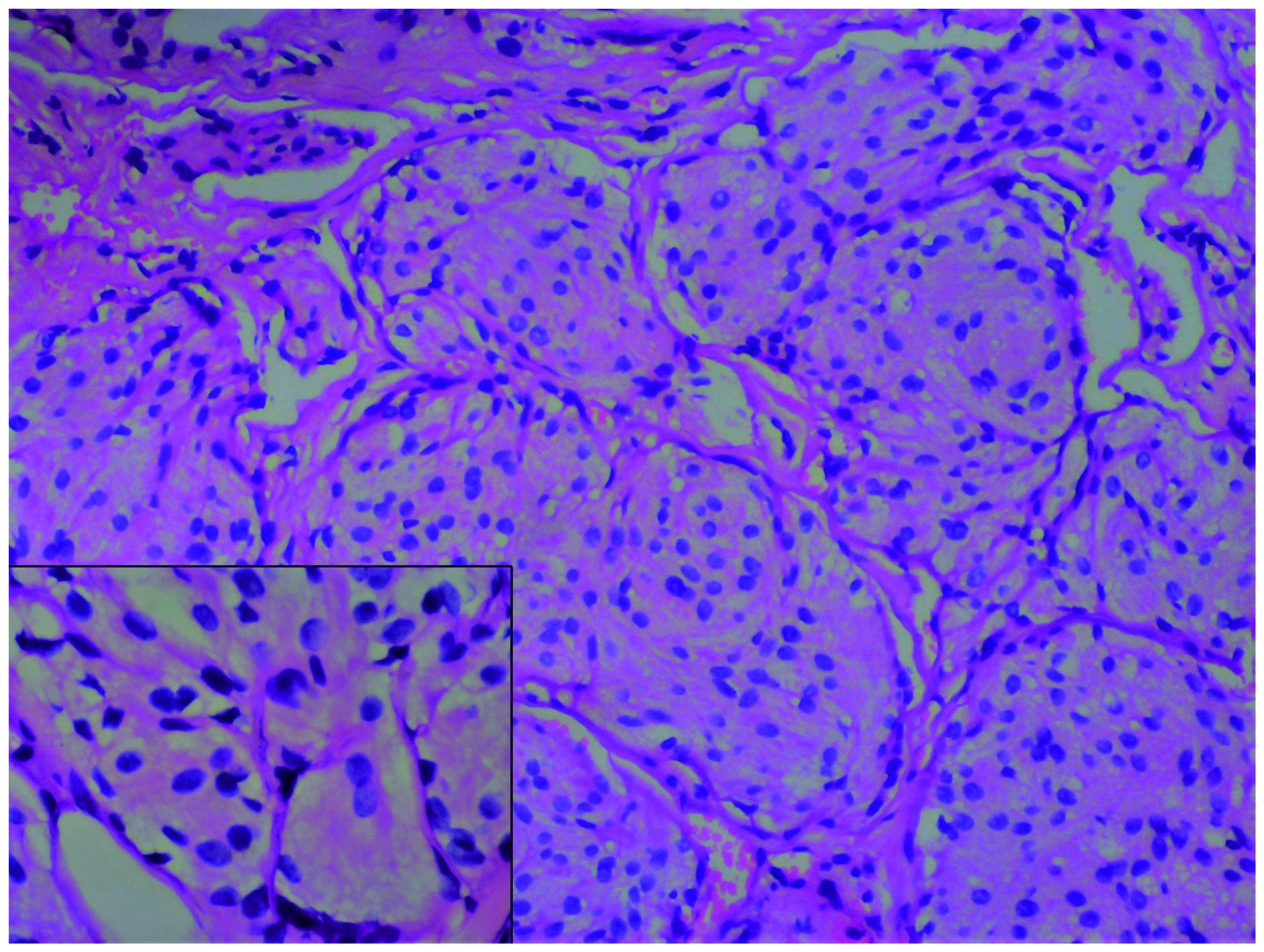
Hematoxylin and eosin-stained sections showing epithelioid cells forming meningiothelial whorls (magnification, ×100). Indistinct cell membranes with uniform nuclei and no mitotic figures (inset; magnification, ×200).

**Figure 3.**
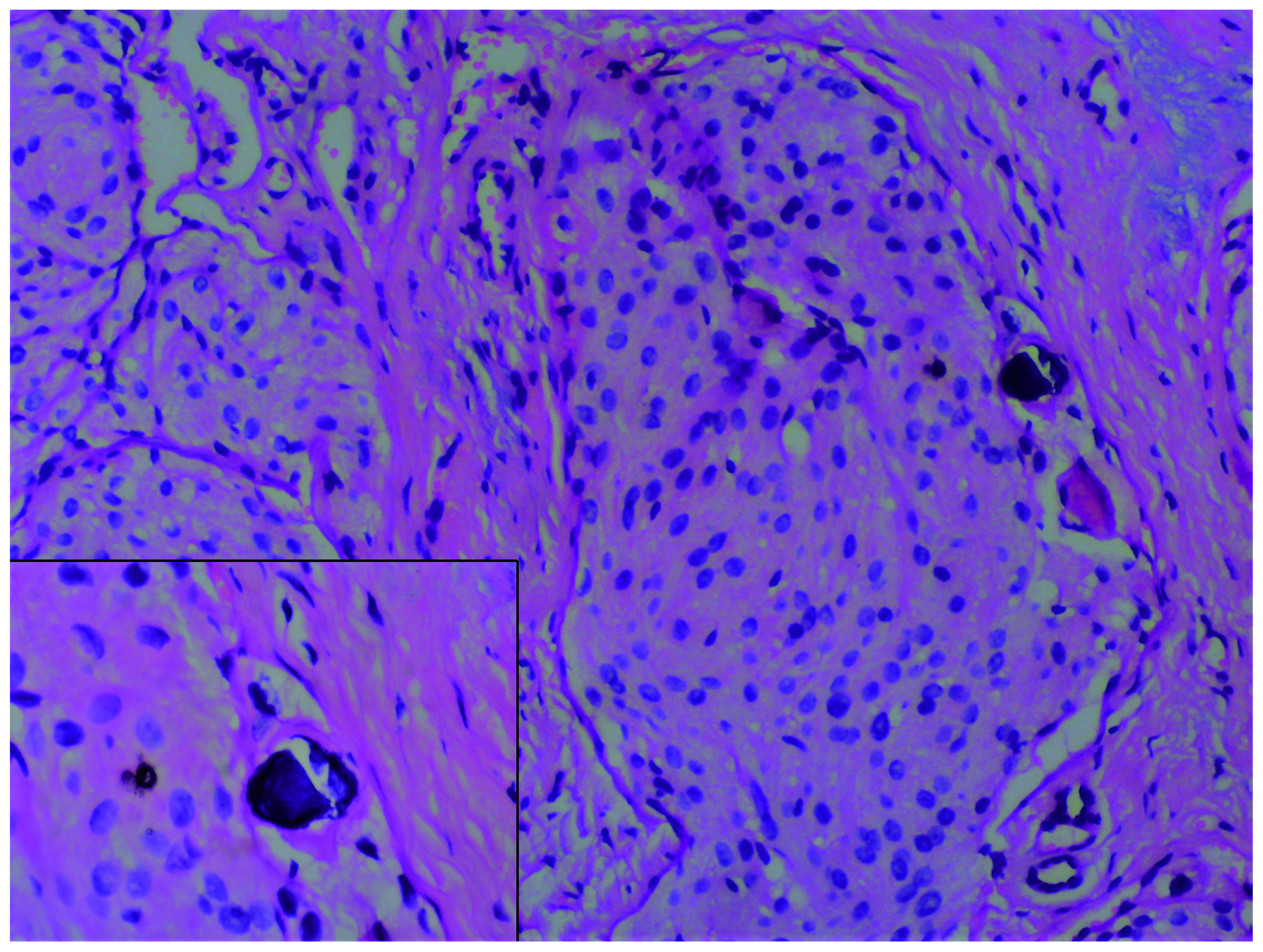
Hematoxylin and eosin-stained sections showing syncytial cells (magnification, ×100). Psammoma bodies seen between meningiothelial cells (inset), (×200).

**Figure 4.**
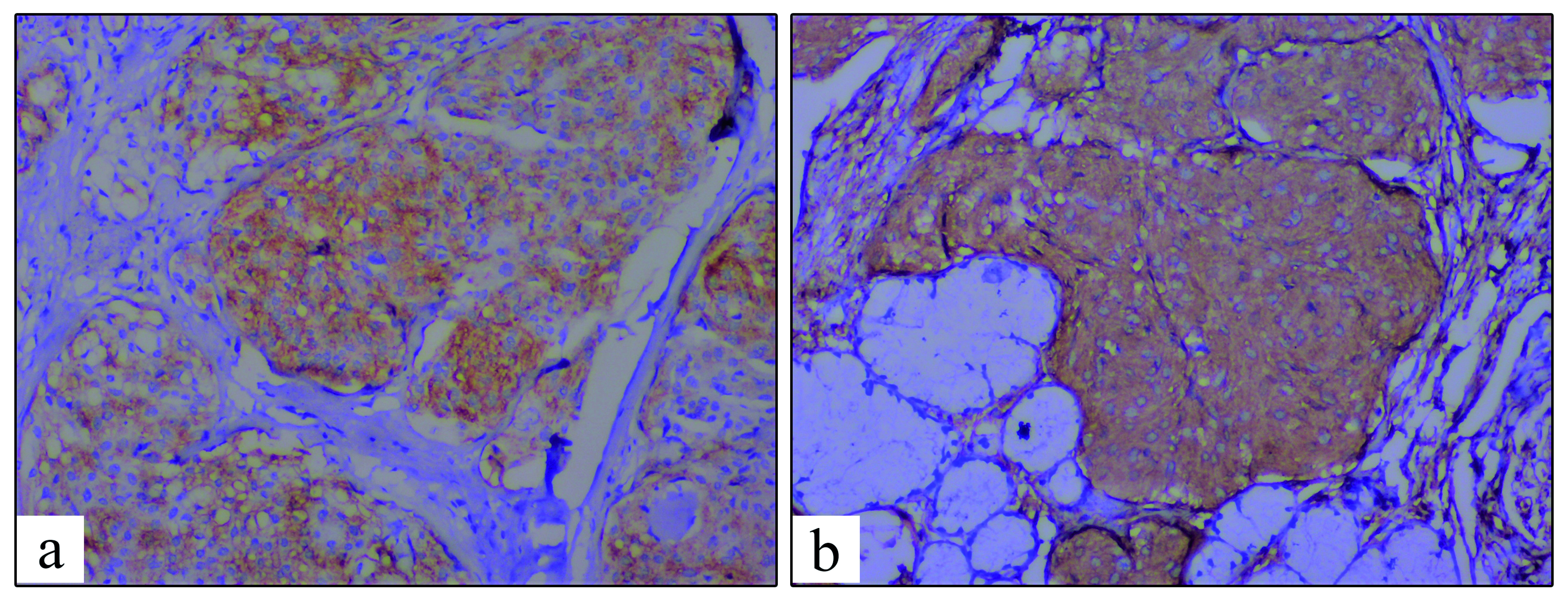
Meningioma tumor cells showing a positive cytoplasmic immunohistochemical reaction for ( **a**) epithelial membrane antigen and ( **b**) Vimentin (magnification, ×200).

**Figure 5.**
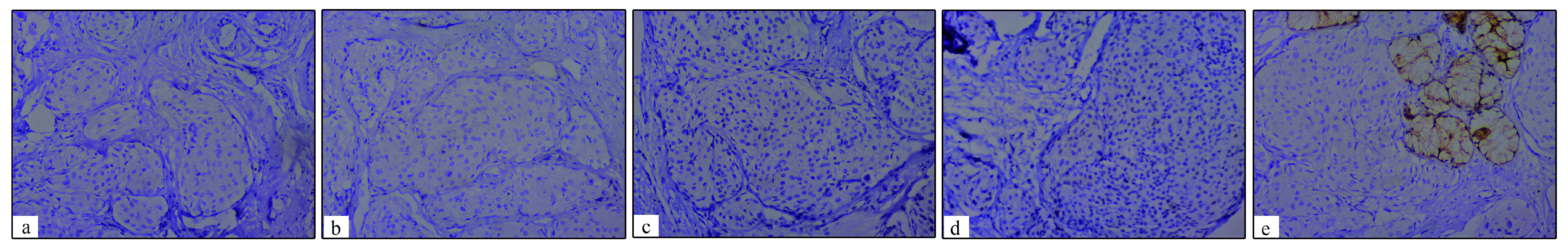
Meningioma tumor cells react negatively following immunohistochemical staining for ( **a**) renal cell carcinoma glycoprotein, ( **b**) S100, ( **c**) chromoginin, ( **d**) p63, ( **e**) PanCK (magnification, ×100).

An incisional biopsy of the lesion was performed. Hematoxylin and eosin stained sections revealed meningothelial cells arranged in lobules. The cells exhibited round to oval nuclei ( Figure 2). Psammoma bodies were also present ( Figure 3). No mitotic activity and no cellular atypia were found. Immunohistochemical staining for tumor-associated markers was performed to confirm the diagnosis of meningioma and to exclude other mimic tumors as metastiatic carcinomas, schwannoma, neurofibroma, paraganglioma and perineurioma. Cells were positively stained using primary antibodies for epithelial membrane antigen (EMA) and vimentin ( Figure 4a, b), but were not stained when using primary antibodies for S100, pancytokeratin, p63, chromogranin and renal cell carcinoma glycoprotein ( Figure 5a–e).

No therapy was administered to the patient during her admission. Unfortunately, the patient did not show up for surgical excision and follow-up.

**Table 1.** Clinicopathological and radiographic data of the documented cases of extracranial meningioma.

Study	Age, years	Gender	Site	Tumor size	Radiographic findings	Treatment	Follow-up
Brown *et al.* ^5^	69	M	Maxilla	NA	ML RL	Not completed	8 years
Simpson and Sneddon ^6^	63	F	Maxillary alveolus	4.5 × 2.7 × 2.7 cm	Well-defined mixed RL RO	Surgical excision.	Under review
Landini and Kitano ^7^	48	F	Mandible	NA	Well-defined RL	Block resection	2 years
Reddi *et al.* ^2^	26	F	Maxilla	3 cm	Ill-defined RL	Surgical excision	2 years
Kishore *et al.* ^8^	44	F	Soft palate	3 × 2 cm	NS	Excisional Biopsy	4 years
Pfeifer *et al.* ^9^	77	F	Maxilla (temporal fossa )	NA	Dense soft tissue mass	Surgical resection	NS
Jones and Freedman ^10^	41	F	Mandible	4 × 2 cm	Well defined RL	Excisional biopsy	NS
Jones and Freedman ^10^	74	F	Mandible	4 × 3 cm	Well-defined RL	Excisional biopsy	NS
Kubotaa *et al*. ^11^	10	M	Mandible	NA	Well-defined RL	Enucleated	4 years
Mussak *et al*. ^12^	62	M	Mandible	7 × 3 cm	Well-defined RL	Segmental mandibulectomy	NS
Lell *et al.* ^13^	40	F	Mandible	NA	Well-defined RL	NS	NS
Mosquede-Taylor *et al.* ^14^	53	F	Mandible	4 cm	Ill-defined mixed RO RL	Surgical excision	6 months
Rushing *et al*. ^15^	NA		Mandible	NA			
Simsek and Komerik ^4^	51	F	Maxilla	2 × 2 cm	Ill-defined mixed RL-RO	Surgical excision	5 years
Pinting *et al.* ^16^	59	M	Maxilla	NA	Well-defined RL	Surgical excision and radiotherapy	NS
Maeng *et al.* ^17^	66	F	Buccal mucosa	2 cm	Heterogenously enhanced mass	Surgical excision	Year and half
Nair *et al.* ^18^	60	F	Buccal mucosa	4 × 3 cm	Mass of heterogeneous density	Surgical resection	One year
Rege *et al.* ^19^	35	M	Mandible	NA	Ill-defined ML RL	Partial resection	5 years
Rommel *et al.* ^20^	20	F	Mandible	2 × 1.8 cm	Well defined RL	No surgical intervention.	One year

M, male; F, female; RL, radiolucent; RO, radioopaque; UL, unilocular; ML, multilocular; NA, not available; NS, not stated.

## Discussion

Primary extra-cranial meningioma is an unusual tumor, especially in the oral cavity ^4^. The first intraoral meningioma reported was by Brown *et al*. in 1976, which presented as a periapical radiolucency in the anterior maxillary region ^5^.

To the best of our knowledge, 19 cases of primary meningioma in the oral cavity have been reported. Of these, 13 were in female patients, which is also true of the present case. However, the age range was wide in the reported cases – between 10 and 77 years old ^2, 4– 20^; in the present case, the patient was 59 years old. Regarding the reported cases of intraoral primary meningioma, 6 of the 18 were in the maxilla ^2, 4– 6, 9, 16^, 10 were in the mandible ^7, 10– 15, 19, 20^, 2 in the buccal mucosa ^17, 18^ and one in soft palate ^8^. To the best of our knowledge, we report the first case in the hard palate.

The histopathological criteria of extracranial meningiomas are similar to those of their intracranial counterparts. All documented cases shared the same characteristics: whorls of spindle cells or epithelioid cell proliferation and psammoma bodies. In our case, diagnosis was challenging because of the tumor’s similarity with other tumor entities of peripheral nerve origin, as well as the uncommon location of the tumor. An immunohistochemical panel of tumor-associated markers was used to confirm the diagnosis and to avoid unnecessary aggressive treatment. Most of the 19 cases reported in the literature were diagnosis using immunohistochemical markers. All reported cases that used immunohistochemistry techniques to diagnose meningioma ^4, 9– 11, 13, 14, 16, 17, 19, 20^ observed that the tumor cells stained positive for monoclonal antibodies against EMA and vimentin, with no immunoreactivity for S-100 protein, which was similar to our findings. However, EMA and vimentin are not useful to differentiate between meningioma and perineuroma as they both express positivity for EMA and vimentin but perineuroma the cells are spindle and elongated however, in our case they are rounded and polyhederal (meningiothelial pattern).

Unfortunately, our patient did not show up for surgical excision and follow-up was not done because of the loss of contact with the patient. However, most of the documented cases were treated successfully without recurrence by surgical excision. Some of the studies, such as that by Rommel *et al*. ^20^, preferred only to follow-up with the patient rather than conduct surgical intervention. However, others preferred to perform aggressive treatment, such as as segmental mandibulectomy or segmental resection ^7, 12^

In conclusion, meningioma is a rare intraoral benign neoplasm. Immunohistochemical markers are an important tool to achieve a final diagnosis, especially for the differentiation from histological mimic entities of peripheral nerve origin, such as perineurioma and neurothekeoma and to avoid unnecessary aggressive treatment. Vimentin and EMA are the two important markers to confirm extra-cranial meningioma diagnosis.

## Data availability

All data underlying the results are available as part of the article and no additional source data are required.

## Consent

Written informed consent for publication of their clinical details and clinical images was obtained from the patient.

## References

[1] El-Naggar AK Chan JK Grandis JR: WHO Classification of Head and Neck Tumours.4 ^th^edition. Lyon: IARC Press,2017 Reference Source

[2] Reddi SP Strauss SI Strauss JE: Anterior maxillary lesion. *J Oral Maxillofac Surg.* 1999;57(10):1234–8. 10.1016/s0278-2391(99)90494-9 10513871

[3] Agaimy A Buslei R Coras R: Comparative study of soft tissue perineurioma and meningioma using a five-marker immunohistochemical panel. *Histopathology.* 2014;65(1):60–70. 10.1111/his.12366 24393170

[4] Simsek H Komerik N: Ectopic Meningioma in the Maxillary Alveolar Ridge: Report of a Case with a Review of the Literature. *IJEDS.* 2012;1(2):98–101. 10.5005/jp-journals-10029-1024

[5] Brown AM Fordham KC Lally ET: Meningioma presenting as an intraoral mass. *Oral Surg Oral Med Oral Pathol.* 1976;41(6):771–6. 10.1016/0030-4220(76)90191-2 1063981

[6] Simpson MT Sneddon KJ: Extracranial meningioma of the oral cavity. *Br J Oral Maxillofac Surg.* 1987;25(6):520–5. 10.1016/0266-4356(87)90146-x 3480004

[7] Landini G Kitano M: Meningioma of the mandible. *Cancer.* 1992;69(12):2917–20. 10.1002/1097-0142(19920615)69:12<2917::aid-cncr2820691209>3.0.co;2-8 1591684

[8] Kishore A Roy D Irvine BW: Primary extracranial meningioma of the soft palate. *J Laryngol Otol.* 2000;114(2):149–50. | 10.1258/0022215001904932 10748837

[9] Pfeifer JD Ashley Hill D Ramos CV: Meningioma Presenting as an Intraoral Mass in a Patient With Neurofibromatosis Type 1. *Arch Pathol Lab Med.* 2000;124(6):898–901. 10835531 10.5858/2000-124-0898-MPAAIM

[10] Jones AC Freedman PD: Primary extracranial meningioma of the mandible: A report of 2 cases and a review of the literature. *Oral Surg Oral Med Oral Pathol Oral Radiol Endod.* 2001;91(3):338–41. 10.1067/moe.2001.112947 11250633

[11] Kubota Y Yamashiro T Kobayashi I: Primary meningioma of the mandible. *Oral Onco Extra.* 2005;41(2):18–21. 10.1016/j.ooe.2004.10.004

[12] Mussak EN Holodny AI Karimi SK: Meningioma of the mandible: imaging with CT. *AJNR Am J Neuroradiol.* 2007;28(6):1157–9. 10.3174/ajnr.A0503 17569978 PMC8134175

[13] Lell M Tudor C Aigner T: Primary intraosseous meningioma of the mandible: CT and MR imaging features. *AJNR Am J Neuroradiol.* 2007;28(1):129–31. 17213439 PMC8134125

[14] Mosqueda-Taylor A Domínguez-Malagon H Cano-Valdez AM: Primary extracranial meningioma of the mandible. *Med Oral Patol Oral Cir Bucal.* 2009;14(4):E167–70. 19333184

[15] Rushing EJ Bouffard JP McCall S: Primary extracranial meningiomas: an analysis of 146 cases. *Head Neck Pathol.* 2009;3(2):116–30. 10.1007/s12105-009-0118-1 19644540 PMC2715454

[16] Pinting L Xiaofeng H Zhiyong W: Extracranial meningioma in the maxillary region. *J Craniofac Surg.* 2013;24(2):e142–4. 10.1097/SCS.0b013e31827c7e9f 23524815

[17] Maeng JW Kim Y Seo J: Primary Extracranial Meningioma Presenting as a Cheek Mass. *Clin Exp Otorhinolaryngol.* 2013;6(4):266–268. 10.3342/ceo.2013.6.4.266 24353870 PMC3863679

[18] Ravindran Nair KS Suresh Babu P Manoj S: The meningioma that got cheeky. *J Maxillofac Oral Surg.* 2016;15(Suppl 2):279–281. 10.1007/s12663-015-0800-7 27408453 PMC4925592

[19] Rege IC Garcia RR Mendonça EF: Primary Extracranial Meningioma: A Rare Location. *Head Neck Pathol.* 2017;11(4):561–6. 10.1007/s12105-017-0813-2 28401439 PMC5677075

[20] Rommel N Bissinger O Rau A: Ectopic meningioma of the mandible in a 20-year-old woman: a case report and literature review. *J Surg Case Rep.* 2017;2017(3):rjx047. 10.1093/jscr/rjx047 28458853 PMC5400473

