# Supplementary material for: Case Report: Rare site for intraoral meningioma
Source: F1000Res. 2020 Apr 6;9:95. Originally published 2020 Feb 7. [Version 2] doi: 10.12688/f1000research.21999.2 (PMC7435328; doi:10.12688/f1000research.21999.2)
Supplement: Peer review history

# Peer review: Reviewer response for version 2

**DOI:** 10.5256/f1000research.25478.r68269

**Published:** 2020-08-18

**Authors:** Hasan Onur Şimşek

**Affiliations:** 1 Department of Oral and Maxillofacial Surgery, Faculty of Dentistry, Aydın Adnan Menderes University, Aydın, Turkey

- Ectopic Meningiomas are seen less frequently in oral regions, so it can be considered as a case report. However, the lack of follow-up of the case reduces the power of the article.
- Clinical data and radiographic findings can be given in more detail.
- Differential diagnoses should be discussed in the discussion section.

Are enough details provided of any physical examination and diagnostic tests, treatment given and outcomes?

Partly

Is the case presented with sufficient detail to be useful for other practitioners?

Yes

Is sufficient discussion included of the importance of the findings and their relevance to future understanding of disease processes, diagnosis or treatment?

Partly

Is the background of the case’s history and progression described in sufficient detail?

Yes

Reviewer Expertise:

maxillofacial surgery

I confirm that I have read this submission and believe that I have an appropriate level of expertise to confirm that it is of an acceptable scientific standard.

# Peer review: Reviewer response for version 2

**DOI:** 10.5256/f1000research.25478.r62034

**Published:** 2020-04-06

**Authors:** Eman Abdelzaher

**Affiliations:** 1 Department of Pathology, Faculty of Medicine, Alexandria University, Alexandria, Egypt

The requested changes were made.

Are enough details provided of any physical examination and diagnostic tests, treatment given and outcomes?

Partly

Is the case presented with sufficient detail to be useful for other practitioners?

Partly

Is sufficient discussion included of the importance of the findings and their relevance to future understanding of disease processes, diagnosis or treatment?

Partly

Is the background of the case’s history and progression described in sufficient detail?

Yes

Reviewer Expertise:

Neuropathology

I confirm that I have read this submission and believe that I have an appropriate level of expertise to confirm that it is of an acceptable scientific standard.

# Peer review: Reviewer response for version 1

**DOI:** 10.5256/f1000research.24261.r59735

**Published:** 2020-03-02

**Authors:** Maha M. Abdelsalam

**Affiliations:** 1 Department of Biomedical Dental Sciences, College of Dentistry, Imam Abdulrahman Bin Faisal University, Dammam, Saudi Arabia

**Case Report: Case Report: Rare site for intraoral meningioma**

 The case report presents an extracranial meningioma of the hard palate. The patient’s gender is conformed to the tumor but there are (likely) no contributing or risk factors associated with its occurrence. The lesion is asymptomatic and incisional biopsy was examined routinely and certain microscopic features were suggestive of meningioma. The specimen was further stained with a panel of markers and was diagnosed as meningioma of the palate. Unfortunately the patient did not return for surgical removal. 

- **Is the background of the case’s history and progression described in sufficient detail?**

The patient’s background can be improved if relevant findings are reported such as previous exposure to radiation as a potential risk for development of meningioma.

- **Are enough details provided of any physical examination and diagnostic tests, treatment given and outcomes?**

This is one of the unfortunate incidents where patient is not committed to treatment.

1. Although it seems that the patient is asymptomatic except for the size of the palatal swelling, absence of radiograph (even if non-indicative) and not mention neurofibromatosis-type2, undermine the reader’s understanding of the case.
2. The clinical picture is good and clear. 

- **Is sufficient discussion included of the importance of the findings and their relevance to future understanding of disease processes, diagnosis or treatment?**

1. Discussion is fine although it would’ve been beneficial to cite the other reported palatal lesions by Kishore A (2000) and Sinha (2002), for example. ^1^^,^^2^
2. The immunohistochemistry test panel discussion would be more useful for the non-pathology reader if there were brief comment on the value of such tests of uncommon oral lesions with perplexing presentations.
3. Consequently, a short list of lesions to be included in a general dentist’s record when similar cases are detected, would be useful.
4. Few editing or English language corrections would help. For example “follow-up was not done because of loose of contact” in which “loss not loose” is meant. The statement is repeated in abstract and discussion and significantly alters the meaning of the sentence.

- **Is the case presented with sufficient detail to be useful for other practitioners?**
  Extracranial meningioma are rare and intraoral lesions are even rarer. It is of high pathological interest of both specialist and general practitioner to get updated on occurrence, presentation and diagnostic methods of such tumors. 

1. The case and the tumor presented are useful, relevant and with corrections, the report would be more impactful.
2. Conclusions must be more powerful and instructive for the practitioner to:
  1. Expect the unexpected when similar lesions are encountered.
  2. Suggest an effective strategy to ensure patient’s return when the outcomes are suspicious.

Are enough details provided of any physical examination and diagnostic tests, treatment given and outcomes?

Partly

Is the case presented with sufficient detail to be useful for other practitioners?

Partly

Is sufficient discussion included of the importance of the findings and their relevance to future understanding of disease processes, diagnosis or treatment?

Partly

Is the background of the case’s history and progression described in sufficient detail?

Partly

Reviewer Expertise:

Oral Pathology, Oral medicine, medical and Dental Education

I confirm that I have read this submission and believe that I have an appropriate level of expertise to confirm that it is of an acceptable scientific standard, however I have significant reservations, as outlined above.

## References

[1] : Primary extracranial meningioma of the soft palate. *J Laryngol Otol*.2000;114(2) : 10.1258/0022215001904932 149-50 10.1258/0022215001904932 10748837

[2] : A solitary huge neurofibroma of the soft palate. *J Laryngol Otol*.2002;116(8) : 10.1258/00222150260171669 637-8 10.1258/00222150260171669 12389695

# Response

**Published:** 2020-03-26

**Authors:** Layla Hafed

**Affiliations:** Department of Oral and Maxillofacial Pathology, Faculty of Dentistry, Ahram Canadian University, Egypt

Firstly, thank you doctor for your valuable comments. We added that data and answers that you commented to the new version of the manuscript. And I will answer them too here as a respond to your report.  

 1- There was no previous exposure to radiation as a potential risk for development of meningioma.This is one of the unfortunate incidents where patient is not committed to treatment.

 2- CT scan was done and there was no bone involvement and  neurofibromatosis-type 2 was excluded because the lesion is single and been present in her oral cavity for 22 year.

 3- Discussion is fine although it would’ve been beneficial to cite the other reported palatal lesions.

 4- The immunohistochemistry test panel discussion was added 

 5- Benign salivary gland neoplasm add to the  list of lesions to be included in a general dentist’s record when similar cases are detected.

 6-  English language corrections were done.

 7- Conclusions  were also corrected to include the consequence of wrong diagnosed.

 Thanks again for your comments which add a lot to our knowledge and for your time too

# Peer review: Reviewer response for version 1

**DOI:** 10.5256/f1000research.24261.r59777

**Published:** 2020-02-25

**Authors:** Eman Abdelzaher

**Affiliations:** 1 Department of Pathology, Faculty of Medicine, Alexandria University, Alexandria, Egypt

The case report is well written and presents an interesting case of ectopic meningioma. Some amendment is needed however.

1. Radiological findings should be included. The authors merely mention that the lesion was not seen by radiology. Please clarify the technique used and relevant findings.
2. The grade of meningioma was not given
3. The differential diagnosis of meningioma from mimics was not sufficiently addressed. Different entities in the differential diagnosis were mentioned at different parts of the article without discussing the differentiating points. And the performed stains would not help in differentiating meningioma from perineurioma, both are positive for EMA and vimentin.
4. Grammatical and spelling mistakes are noted here. 

Are enough details provided of any physical examination and diagnostic tests, treatment given and outcomes?

Partly

Is the case presented with sufficient detail to be useful for other practitioners?

Partly

Is sufficient discussion included of the importance of the findings and their relevance to future understanding of disease processes, diagnosis or treatment?

Partly

Is the background of the case’s history and progression described in sufficient detail?

Yes

Reviewer Expertise:

Neuropathology.

I confirm that I have read this submission and believe that I have an appropriate level of expertise to confirm that it is of an acceptable scientific standard, however I have significant reservations, as outlined above.

# Response

**Published:** 2020-03-26

**Authors:** Layla Hafed

**Affiliations:** Department of Oral and Maxillofacial Pathology, Faculty of Dentistry, Ahram Canadian University, Egypt

Thank you Dr. Eman for your valuable comments and for your time. We added that data and answers that you commented to the new version of the manuscript. And I will answer them  here too as a respond to your report.  

1-  All the English language corrections were done.

2- CT scan was done and there was no bone involvement.

3- Meningioma grade I.

4- The list of differential diagnosis was done and discussed.
